# Mate and fuse: how yeast cells do it

**DOI:** 10.1098/rsob.130008

**Published:** 2013-03

**Authors:** Laura Merlini, Omaya Dudin, Sophie G. Martin

**Affiliations:** Department of Fundamental Microbiology, Faculty of Biology and Medicine, University of Lausanne, Biophore building, Lausanne 1015, Switzerland

**Keywords:** mating, yeast, pheromone, polarization, mitogen-activated protein kinase (MAPK), Cdc42, cell fusion

## Abstract

Many cells are able to orient themselves in a non-uniform environment by responding to localized cues. This leads to a polarized cellular response, where the cell can either grow or move towards the cue source. Fungal haploid cells secrete pheromones to signal mating, and respond by growing a mating projection towards a potential mate. Upon contact of the two partner cells, these fuse to form a diploid zygote. In this review, we present our current knowledge on the processes of mating signalling, pheromone-dependent polarized growth and cell fusion in *Saccharomyces cerevisiae* and *Schizosaccharomyces pombe*, two highly divergent ascomycete yeast models. While the global architecture of the mating response is very similar between these two species, they differ significantly both in their mating physiologies and in the molecular connections between pheromone perception and downstream responses. The use of both yeast models helps enlighten both conserved solutions and species-specific adaptations to a general biological problem.

## 2. Introduction

**Figure 1.**
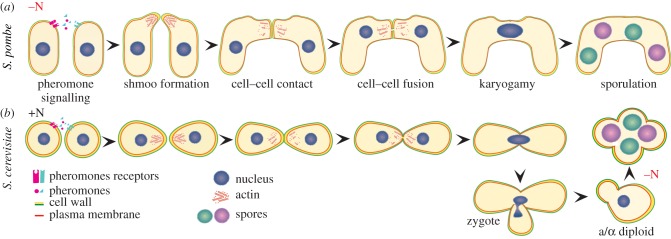
Sequential steps during mating in *Schizosaccharomyces pombe* and *Saccharomyces cerevisiae*. (*a*) In fission yeast, the mating process is triggered by nitrogen starvation when compatible partners are present. (*b*) Budding yeast cells of opposite mating type can instead mate spontaneously on rich medium to form stable diploids that undergo sporulation upon starvation. In both organisms after pheromone exchange, cells grow in a polarized manner in the direction of their partner and undergo fusion, karyogamy and sporulation. See text for details.

Cell polarization induced by external signals is a fundamental cellular property that relies on cytoskeletal and membrane re-organization in response to specific cues. Many cell types exhibit chemotaxis or chemotropism in response to external signals, which are essential for functions as diverse as neuronal pathfinding, wound healing or pathogenesis. Unicellular yeast models are potent systems to understand the molecular interactions that generate cell polarity induced by external inputs. Indeed, yeast cells exhibit chemotropism in response to pheromones produced by partner cells during the mating process. Pheromones are recognized by specific receptors expressed on the surface of cells of the opposite mating type and this binding stimulates the activation of receptor-associated heterotrimeric G-proteins, which in turn promote the activation of a conserved mitogen-activated protein kinase (MAPK) module. By ultimately activating a specific transcription factor, MAPK cascade components modulate the expression of mating-specific genes, thus promoting cell cycle arrest, polarized morphogenesis in the direction of the partner cell (a process known as shmooing), cell–cell fusion and karyogamy to produce a diploid zygote (figure 1).

The aim of this review is to give an overview of the mating process of the two distantly related ascomycete ‘cousins’, the yeast models *Schizosaccharomyces pombe* and *Saccharomyces cerevisiae*. It should be highlighted here that these two yeasts are in fact highly divergent, with an evolutionary distance estimated at close to 1 Gyr [1,2]. We will focus on the spatial reorganization of the cell for zygote formation, showing how yeast cells re-orient their growth in the direction of a pheromone source and describing the connections between pheromone signalling and cell polarization. We will also survey the complex mechanisms that allow cells to fuse. By describing the mating process of the two yeast models, we will direct our attention to what it is already known, but also speculate about open questions that would be useful to address in the future. For sake of clarity, proteins will as much as possible be described by their generic function. Their organism-specific names are listed in table 1. Finally, we will look at the implications that the study of yeast mating could have for the understanding of analogous fundamental biological processes in higher eukaryotes.

**Table 1.** Mating and fusion pathway components in budding and fission yeast. Despite confusing nomenclature, most proteins involved in mating signalling and shmoo formation are conserved between *S. cerevisiae* and *S. pombe*. However, fission yeast cells notably lack homologues of the scaffold proteins Ste5 and Far1, and a Gγ subunit has not yet been identified. A more distantly related Ras-like protein, Rsr1/Bud1, also plays important roles during vegetative cell polarization in budding yeast. Some components of the fusion process are also conserved. However, despite the essential role of the formin Fus1 in *pombe* cell–cell fusion, the possible roles of the *cerevisiae* formins Bnr1 and Bni1 in fusion are unclear. Conversely, the two budding yeast *FUS* genes do not have orthologues in fission yeast. See text for details.

generic name/function	*S. cerevisiae*	*S. pombe*
**SIGNALLING**		
pheromones	a-factor, α-factor	P-factor, M-factor
G-protein coupled receptors	Ste3, Ste2	Mam2, Map3
G-protein α subunit	Gpa1	Gpa1
G-protein β subunit	Ste4	Gnr1 (putative)
G-protein γ subunit	Ste18	unknown
PAK kinase	Ste20	Shk1
MAPK scaffold	Ste5	no homologue
other MAPK scaffold	Ste50	Ste4 (putative)
MAPKKK	Ste11	Byr2
MAPKK	Ste7	Byr1
MAPK	Fus3, Kss1	Spk1
transcription factor	Ste12	Ste11
scaffold for shmoo orientation	Far1	no homologue
Cdc42 GTPase	Cdc42	Cdc42
Cdc42-GEF	Cdc24	Scd1
Cdc42-scaffold	Bem1	Scd2
Ras GTPase^a^		Ras1
Formin	Bni1, Bnr1	For3?
**FUSION**		
Prm1 (4-pass transmembrane protein)	Prm1	Prm1
other 4-pass transmembrane proteins	Fig1	Dni1
transmembrane protein	Fus1	no homologue
Rho-GEF	Fus2	no homologue
Formin	Bni1, Bnr1?	Fus1
type V myosin	Myo2	Myo51, Myo52?
tropomyosin	Tpm1	Cdc8

^a^Ras1 has an essential role in mating in fission yeast, whereas its budding yeast counterparts, Ras1 and Ras2, are implicated in a distinct, cAMP signalling pathway.

## 3. Mating signalling and polarization

At first glance the overall process of mating appears quite similar in the two yeast models. Indeed, in both cases peptide pheromones are recognized by G-protein coupled receptors expressed on the cell surface. The receptors have a conserved structure with seven transmembrane domains, a cytoplasmic C-terminal tail mediating desensitization and pheromone-induced internalization, and an intracellular loop involved in G-protein binding. Moreover, in both cases the signal is transmitted by MAPKs to a transcription factor that activates the expression of mating-specific genes. However, a more detailed analysis reveals many differences between the two species, which is perhaps not surprising given their long evolutionary distance.

### 3.1. Activation of mating signalling in *Saccharomyces cerevisiae*

**Figure 2.**
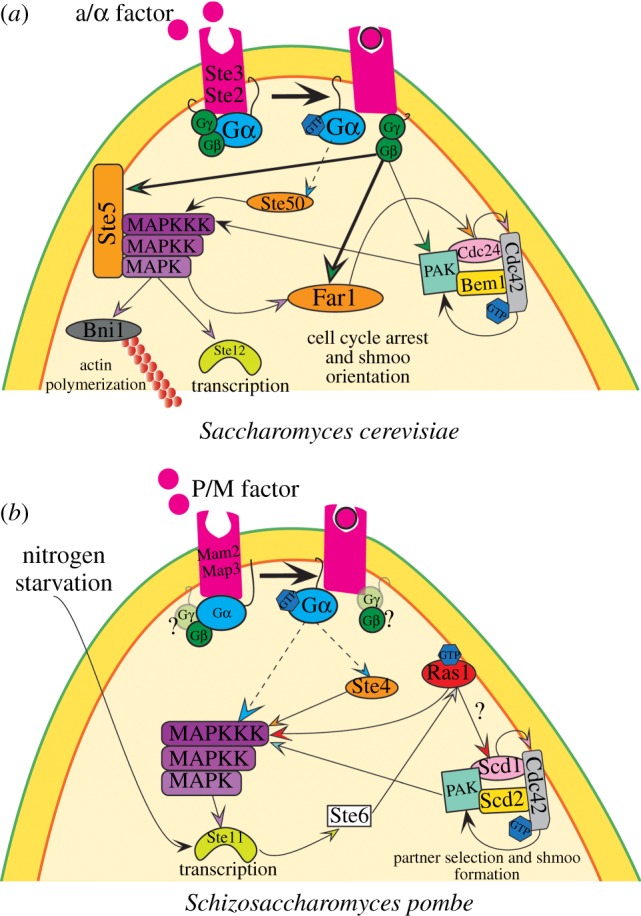
Mating signalling in budding and fission yeast. Pheromone binding to its G-protein coupled receptor leads to Gα activation (Gα-GTP) and dissociation from the Gβγ heterodimer, and activation of a conserved MAPK cascade that leads to the transcription of mating-specific genes, cell polarization in the direction of partner cells and subsequent fusion of mating pairs. (*a*) In budding yeast, the signal is transmitted by the Gβγ dimer, through Gβ interactions with several effectors. Notably Gβ regulates the activity of two distinct scaffold proteins to activate the conserved MAPK (through Ste5) and Cdc42 (through Far1) modules. (*b*) In fission yeast, the transcription factor Ste11 is activated upon nitrogen starvation and regulates the expression of essential signalling genes, such as the Ras1-GEF Ste6. Gα is responsible for signal transmission in this organism and appears to activate the MAPK cascade (directly or indirectly) cooperatively with Ras1 and the scaffold Ste4. Dashed arrows indicate hypothetical interactions; question marks indicate components not yet identified (*pombe* Gγ) or interactions not specifically demonstrated during mating (Ras1–Scd1). See text for details.

The mating process has been extensively studied in *S. cerevisae* over the last 30 years. At the physiological level, budding yeast cells mate spontaneously on rich medium when in the presence of cells of the opposite mating type, forming stable diploids, which sporulate upon starvation (figure 1*b*). Pheromones (called a- and alpha-factor) are captured by the receptors Ste3 and Ste2 (for a- and alpha-factor, respectively), which activate the same Gαβγ heterotrimeric G-protein. Pheromone binding stimulates GDP to GTP exchange on the Gα subunit (Gpa1), which allows the released Gβγ (Ste4 and Ste18) heterodimer to activate mating signalling [3] (figure 2*a*). In particular, Gβ directly interacts with key effectors: in the presence of pheromones, Gβ binds to the p21-activated kinase (PAK)-like kinase Ste20 [4], the MAPK scaffold protein Ste5 [5], the Cdc42-guanine-nucleotide exchange factor (GEF) Cdc24 [6–8] and the scaffold protein Far1 [9] (figure 2*a*).

The central hub for mating signalling is Ste5. This scaffold protein serves to link the Gβ with the PAK kinase Ste20 and the MAPK module, and has an essential role in promoting MAPK cascade activation [10,11]. The PAK kinase is the upstream component of the MAPK cascade, and activates the downstream kinases Ste11 (MAPKKK), Ste7 (MAPKK) and Fus3 or Kss1 (MAPK) [12] (figure 2*a*). After pheromone stimulus, the Ste5 scaffold is rapidly translocated to the plasma membrane by Gβγ [13,14], where it initiates and amplifies mating signalling [15]. Ste5 membrane binding additionally depends on two membrane-binding regions, an N-terminal amphipathic helix and a PH domain [16,17]. Ste5 also binds the Cdc42 GEF Cdc24, which may contribute to its re-localization to the cell cortex [7]. At the cortex, Ste5 simultaneously binds all the components of the MAPK module through distinct domains [18] and acts as a cofactor by increasing the low MAPKK Ste7 intrinsic phosphorylation activity on MAPK Fus3 [19]. Membrane binding also relieves an auto-inhibitory interaction in Ste5 to promote Fus3 activation [20]. Finally, Ste5, by binding to the phosphatase Ptc1, also promotes a switch-like activation of Fus3 [21]. Once activated, Fus3 dissociates from Ste5 and serves to activate the transcription factor Ste12 [22,23]. Active Fus3 phosphorylates three additional targets: the cyclin inhibitor Far1 [24,25] and the cyclin-dependent kinase Cdk1 (Cdc28) [26] to promote cell cycle arrest in G1 phase [27], and the formin Bni1 to regulate actin polarization and cell fusion [28].

### 3.2. Polarizing growth towards the partner cell in *Saccharomyces cerevisiae*

Budding yeast cells are exquisitely able to project a shmoo towards the source of a pheromone gradient, allowing them to grow towards a potential mating partner. Early important experiments showed that, in mating mixtures of *MAT***a** cells containing the same number of pheromone-producing and non-pheromone-producing *MAT*α partners, *MAT***a** cells are able to discriminate between the two categories, and mate almost exclusively with pheromone-producing partners [29]. Nevertheless, when pheromone receptors are saturated through high isotropic concentrations of pheromone, cells get confused and mate randomly with either pheromone-producing and non-producing partners, through the so-called ‘default pathway’ [30], where the presumptive bud site becomes the shmoo site [31]. In addition to producing pheromones, yeast cells also produce proteases that cleave and inactivate pheromones, thus actively remodelling the pheromone landscape in their environment. In particular, the alpha-factor protease Bar1, which is released by *MAT***a** cells, helps these cells avoid each other [32,33]. Simplified setups, such as release of pheromone through micropipette or microfluidic devices, have been used to show that *MAT***a** cells orient growth towards the source of an artificial pheromone gradient [34–37]. Yeast cells generally initiate shmoo growth towards the gradient source, but are also able to adjust their shmoo trajectory during polarized growth [36,37]. This directional correction is probably due to polarization factors undergoing a random walk at the plasma membrane biased by receptor-activated Gβγ association [38].

The main regulator of cell polarization is the small GTPase Cdc42. Its role in symmetry breaking to define the site of bud emergence during mitotic growth has been extensively studied [39–41]. Cdc42 is activated by a single GEF Cdc24, which is positively regulated by the scaffold protein Bem1 [42]. In the absence of other cues in vegetative growing cells, Bem1, which binds Cdc42, its GEF and a PAK kinase, functions in a positive feedback loop to break symmetry by enforcing the formation of a single cluster of active Cdc42 [43–45] (figure 2*a*). During mating, Cdc42 regulates the PAK kinase Ste20 localization to the plasma membrane and its activation; indeed active Cdc42 (Cdc42-GTP) binds Ste20 and stimulates its kinase activity [46]. Consistently, mutations impairing Cdc42 activity or its GEF affect pheromone-induced MAPK signalling in budding yeast [8,47]. Like the PAK kinase, the Cdc42 GEF Cdc24 interacts with Gβ [6,8]. However, *in vivo* this interaction depends on the adaptor protein Far1 [9,48] and is required for the localized activation of Cdc42.

Far1, a scaffold structurally similar to Ste5 [17,49,50], has a fundamental role in determining the site of cell polarization during mating [51]. In vegetative growing cells, Far1 sequesters Cdc24 in the nucleus during mitosis, and Far1 degradation is required for Cdc24 release and recruitment to the incipient bud site in late G1 phase [52]. Nevertheless, during mating a Far1–Cdc24 complex can translocate from the nucleus to the cell cortex, where it interacts with Gβγ and recruits Cdc42 and Bem1 away from the bud site, thus providing the switch from bud growth to shmoo growth [6,9,50,53]. The disruption of *far1* does not affect the ability of cells to shmoo *per se*, but impairs the displacement of polarity factors from the site of bud emergence, thus leading to the formation of a mislocalized shmoo at the bud site. Consistently, mutations that prevent the formation of a Cdc24–Far1–Gβγ complex prevent the correct orientation of shmoos towards a pheromone source [6,9,48]. In addition to Far1, the scaffold protein Bem1 can also bind the PAK kinase Ste20 and the MAPK scaffold Ste5 [54], to recruit components of the MAPK pathway to the shmoo site. Through these interactions, Bem1 potentiates the MAPK cascade, leading to a local amplification of the signal [55]. Active Cdc42 then promotes actin assembly, resulting in polarized growth.

The Cdc24–Far1–Gβγ complex is not the only molecular connection between pheromone receptors and the polarization machinery. Gα also has a positive role in promoting chemotropism in budding yeast. Indeed, Gα directly interacts with active (phosphorylated) Fus3 MAPK, thus promoting its recruitment to the shmoo site [56]. Gα also promotes Fus3 recruitment in an indirect way: Gα binds the RNA-binding protein Scp160 [57], which, upon pheromone treatment, interacts with polarity and mating-specific mRNA, including *fus3* mRNA, thus ensuring its subsequent translation and enrichment at the shmoo site [58]. This results in a gradient of active Fus3 from the shmoo tip, which was proposed to be important to maintain a local pool of activity [59]. Consistently, active Fus3 at the shmoo site phosphorylates and stably localizes the formin Bni1 [28] and also phosphorylates Gβ, thus stabilizing the Far1–Gβγ complex [56]. In turn, the formin Bni1, by assembling actin cables, contributes to the polarized recruitment of the MAPK scaffold Ste5, the Cdc42 GEF Cdc24 and Fus3 itself for efficient Fus3 activation [60], as well as to the delivery of vesicles that promote wandering of the polarization patch for shmoo re-orientation [38]. In sum, during budding yeast mating, several mechanisms cooperate to link pheromone signalling with cell polarization, and the molecular components required for shmoo orientation are well defined. However, the mechanisms by which Cdc42 becomes initially asymmetrically localized in response to a pheromone gradient remain unclear.

### 3.3. Physiological and molecular differences for mating in *Saccharomyces cerevisiae* and *Schizosaccharomyces pombe*

Despite superficial similarities between the mating processes of *S. cerevisiae* and *S. pombe*, which we will describe below, these organisms exhibit major differences. The first lies in their distinct physiologies for sexual differentiation: while *S. cerevisiae* mates spontaneously and forms stable diploids, sexual differentiation in *S. pombe* is triggered by starvation, and the diploid cells formed are unstable, ensuring a strict coupling between mating and sporulation (figure 1). Second, whereas signalling downstream of the pheromone receptors is principally transmitted through Gβγ released from Gα inhibition in *S. cerevisiae*, it is transmitted through activated Gα in *S. pombe* [61]. Finally, *S. pombe* cells lack homologous genes to either Ste5 or Far1 scaffolds [49], but rely on the function of a Ras GTPase for both signalling and cell polarization [62], indicating that the molecular connections between pheromone sensing, signalling and polarization are distinct in the two species (figure 2).

### 3.4. Activation of mating signalling in *Schizosaccharomyces pombe*

In fission yeast, sexual differentiation is triggered by starvation when compatible mating partners are present. This leads to arrest in G1 phase of the cell cycle, mating-type-specific pheromones and pheromone receptor production, polarized growth in the direction of pheromone source, fusion of mating partners, karyogamy, meiosis and formation of resistant spores [63] (figure 1*a*). Upon nitrogen starvation, the transcription factor Ste11 (not to be confused with its *S. cerevisiae* homonym) is activated in three different ways [64]. First, lack of nitrogen leads to the inactivation of TORC1 and cAMP pathways, both of which repress *ste11* expression during vegetative growth [64,65]; second, nutrient starvation promotes the activation of the stress-responsive MAPK pathway, which enhances *ste11* expression [66]; and finally the mating-pheromone responsive MAPK pathway also induces Ste11 when pheromone binds to its receptor [67]. Ste11 acts as a developmental switch. Indeed, the expression of its targets induces physiological and morphological changes that lead to sexual differentiation, and its constitutive expression causes starvation-independent sexual differentiation [68]. Notably, Ste11 activates pheromone signalling, by directly stimulating pheromone production and pheromone receptor expression [63]. As Ste11 both activates pheromone signalling and is induced by it, it provides a positive feedback for the mating response, where pheromone signalling components cooperate with Ste11 itself, to enhance their own expression and to promote the transcription of other Ste11-dependent genes [67,69].

Pheromones (P- and M-factors, produced by *h^+^* and *h^–^* cells, respectively) are bound by the receptors Mam2 and Map3 (for P- and M-factor, respectively), which are presumably coupled to the same components of a still incomplete heterotrimeric G-protein. Here, the Gα protein Gpa1 is responsible for the activation of the MAPK pathway [61] (figure 2*b*). It is, however, unknown whether there exists a Gβγ dimer that negatively regulates Gα: a putative Gβ subunit, Gnr1, interacts with Gpa1 in a two-hybrid assay and may inhibit Gα-mediated signalling [70], but whether it acts as a monomer or coupled to an unidentified Gγ remains unclear. Notably, in *S. cerevisiae*, ‘kelch repeat’ proteins were shown to mimic Gβ subunits and to inhibit the Gα protein Gpa2, which regulates invasive growth response and filamentous differentiation in the absence of Gγ [71]; and in *Kluyveromyces lactis* Gβ subunit alone is able to positively activate the mating pathway in the absence of Gγ [72]. Once activated, Gα signals to the MAPK cascade, which consists of the MAPKKK Byr2, the MAPKK Byr1 and the MAPK Spk1 [73,74] (figure 2*b*). Spk1 was shown to directly target the transcription factor Ste11, thus promoting its activation [75].

So far no data indicate a direct interaction between the Gα and the MAPKKK Byr2, and it is also possible that unknown scaffold or linker proteins mediate Byr2 activation, although there exists no Ste5 homologue. One promising candidate is the mating-specific protein Ste4, essential for sexual differentiation [76], which interacts with Byr2 and promotes its activation [77–79]. Ste4 (not to be confused with its *S. cerevisiae* homonym) shows homology to budding yeast Ste50 [77], a protein involved in the activation of the MAPKKK Ste11 in *S. cerevisiae* [80]. Interestingly, a Ste50 homologue binds both MAPKKK and the Gα protein in *K. lactis*, an ascomycete closely related to *S. cerevisiae* [81], and is necessary for mating signalling in *Cryptococcus neoformans*, a basidiomycere species that lacks a Ste5 homologue [49,82], thus supporting the idea that Ste4 may link Gα with the MAPKKK Byr2.

The small GTPase Ras1, the only homologue of human Ras in fission yeast, is another regulator of the MAPK cascade [62,74,83,84]. Differently, its budding yeast homologues do not participate in mating: indeed, Ras1 and Ras2 are implicated in cell proliferation by regulating adenylate cyclase activity [85], whereas a second Ras-related small GTPase, Rsr1/Bud1, is critical for bud-site selection and polarity establishment through interaction with Cdc42 and its GEF Cdc24 [86]. During mating in fission yeast, Ras1 is activated at the cell cortex by the GEF Ste6, which promotes GDP to GTP exchange, and inactivated by the GTPase-activating protein (GAP) Gap1 [87]. *ste6* is not expressed during vegetative growth, because its transcription is regulated by Ste11 [88] (figure 2*b*). Both Ste6 and Ras1 are essential for sexual differentiation [83,84,89]. For MAPK activation, Ras1 was proposed to regulate the localization of Byr2 MAPKKK to the plasma membrane [90,91]. Because both Ras1 and Ste4 are essential for mating and bind Byr2 through distinct domains [77–79], both proteins may synergize for Byr2 activation. Finally, Cdc42 signalling may also contribute to MAPK activation, as one Cdc42 effector, the essential PAK kinase Shk1, promotes the transition of the MAPKKK Byr2 to an activated state [78] (figure 2*b*). Surprisingly, however, the Cdc42 GEF Scd1 and the scaffold protein Scd2 (Bem1 homologue), which promote Cdc42 activation during mating and are essential for the mating process, are not required for MAPK activation [62]. One possibility is that residual Cdc42 activity (through the action of a second GEF, Gef1) may be sufficient for activation of Shk1, but not for polarized cell growth, resulting in sterility. In sum, several components were found to promote Byr2 MAPKKK activation, some of which are induced by MAPK signalling and provide a positive feedback that reinforces pheromone signal, but the molecular links with the Gα remain unknown.

### 3.5. Polarizing growth towards the partner cell in *Schizosaccharomyces pombe*

As in budding yeast, Cdc42 is the major cell polarity regulator. Bendezú and Martin [92] have recently shown that during mating an active Cdc42 complex samples the cell periphery before specifying and stably localizing at the shmoo tip. Cdc42, Scd1 and Scd2 form dynamic zones, which explore the cell periphery in early stages of mating in response to low-level pheromone signalling. During dynamic exploration, cell wall synthases Bgs1 and Bgs4, which are required for growth, are retained in endomembranes and co-localize with Cdc42 only upon partner cell choice. This dynamic exploration is required for orientation of the mating projection, as mutants that constitutively activate pheromone signalling prevent this dynamic exploration and lead to the default choice of a cell pole for growth. Conversely to wild-type strains, these mutants preferentially mate with sister cells, suggesting that Cdc42 exploration is important for partner selection [92]. This phenotype is reminiscent of that of *far1* mutants in *S. cerevisiae*, which shmoo from bud site landmarks by default in the absence of orientation information [9,48,51], but whether *far1* mutations increase the relative choice for sister cells has not been studied yet. However, no Far1 homologue exists in *S. pombe*, such that the mechanisms that promote the recruitment of active Cdc42 to pheromone-bound receptors are unknown.

In addition to its role in MAPKKK activation, Ras1 was also proposed to promote Cdc42 by activating the Scd1 GEF [93]. Indeed, *ras1Δ* cells are almost round-shaped, even during vegetative growth [83]. For this function, Ras1 is activated by a second, constitutively expressed Ras1-GEF, Efc25, which in contrast to the Ste6 GEF is required for cell morphology but not for mating [94]. Strikingly, Ras1 was shown to localize to both plasma and endomembranes, with manipulations restricting localization to a single membrane leading to either sterility or morphology defects during mitotic growth [95]. Together with the study of Ras1 GEFs, these data were collectively interpreted as two Ras1 pools insulated from each other by virtue of their distinct localization, one on endomembranes activated by the Efc25 GEF and regulating the Cdc42 GEF Scd1, the other at the plasma membrane activated by the Ste6 GEF and regulating the Byr2 MAPKKK [94,95]. However, the observations that (i) deletion of *efc25* has no effect on mating [94], while deletion of *scd1* causes sterility [62], and (ii) a plasma membrane-restricted Ras1 allele, which displayed abnormal morphology during vegetative growth [95], was nevertheless fertile and thus must have successfully activated Scd1 for mating, suggest distinct interpretations: either Scd1 is activated in a Ras1-independent manner during mating, or distinct pools of Ras1 control Scd1 in vegetative and mating cells. In support of this second hypothesis, Ras proteins are directly involved in chemotaxis in the social amoeba *Dictyostelium discoideum*. Indeed, in this organism, active Ras proteins localize at the leading edge of migrating cells upon stimulation and drive cell motility [96], suggesting that *S. pombe* Ras1 could mediate Cdc42-dependent cell polarization also during fission yeast mating. Whether Ras1 may play a role in linking the polarization machinery to pheromone sensing is an interesting possibility that remains to be explored.

One interesting question is why the *Schizosaccharomyces* lineage lost Ste5 and Far1 scaffolds: at least one scaffold is present in basidiomycetes and ascomycetes, except for the *Schizosaccharomyces* lineage [49]. While future dissection of the molecular connections between pheromone sensing, signalling and polarization may provide answers to this question, a possible interpretation may lie in the distinct physiologies of the two yeasts. One important function of the Ste5 scaffold in *S. cerevisiae* is to insulate the mating-specific MAPK cascade from other MAPK cascades, in particular the one activated upon starvation, which shares identical components [20,97]. As starvation and mating are tightly coupled in the fission yeast, and no component of the mating MAPK cascade is shared with other pathways, such insulation may have become dispensable. Similar reasoning may be applied to Far1. Besides its role in mating projection orientation, Far1 is an essential cyclin inhibitor, keeping cells in G1 phase [24]. Starvation may promote G1 arrest through alternative mechanisms in fission yeast, which may have rendered Far1 dispensable. It is, however, noteworthy that pheromones also promote cell cycle arrest in *pka1Δ* mutant cells unable to sense nutrients, although through unknown mechanisms [92,98,99].

## 4. Fusion of the mating partners

**Figure 3.**
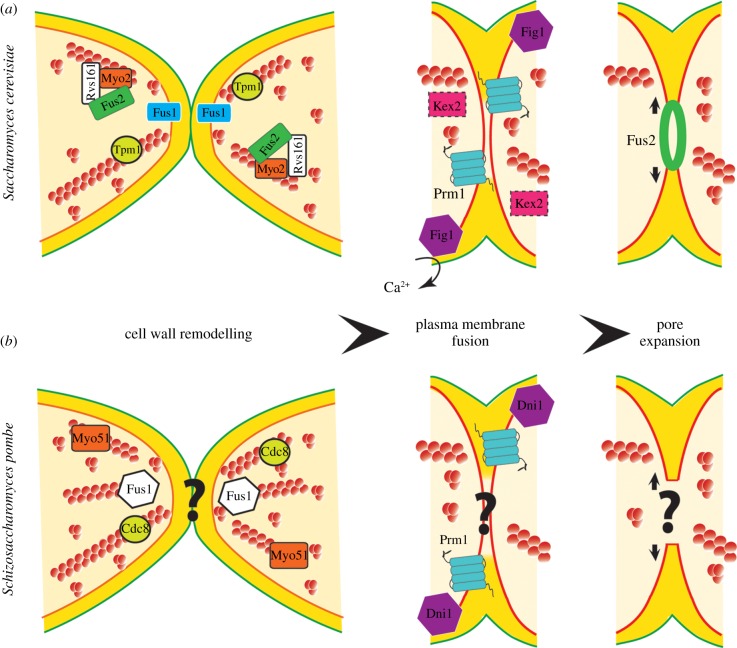
Cell–cell fusion in *S. cerevisiae* and *S. pombe*. Upon cell–cell contact, cell wall remodelling allows plasma membrane contact, fusion pore formation and pore expansion for zygote formation. (*a*) In budding yeast, Fus1 and Fus2 are implicated in cell wall remodelling. Transmembrane protein Fus1 localization to the fusion site depends on active Cdc42 and vesicle trafficking. Fus2 is transported along actin cables and needs Myo2 and Rvs161 for its proper localization. Additionally, Fus2 localizes as a ring later during fusion and was proposed to participate in pore expansion. The conserved transmembrane protein Prm1 and transmembrane proteins Kex2 and Fig1 cooperate for plasma membrane fusion. (*b*) In fission yeast, Fus1 is a formin essential for fusion, with tropomyosin Cdc8 and myosin V Myo51 also implicated. The only protein so far implicated in plasma membrane fusion in this organism is Dni1. The possible role of Prm1 has not yet been described. Please note that budding yeast and fission yeast Fus1 proteins are completely unrelated. See text for details.

The purpose of the mating process is to permit the fusion of the two haploid partner cells in order to produce a diploid zygote. Cell fusion requires two main steps: first, the cell walls at the contact site are remodelled to form a continuous structure joining the two cells together and called the pre-zygote, which is then degraded to permit plasma membrane contact. Second, one or several fusion pores are likely to form and expand to fuse the adjoining plasma membranes together (figure 3). Owing to high internal turgor pressure, these two steps need to be carefully coordinated to prevent cell lysis. Upon cell membrane fusion, the nuclei come in contact and undergo karyogamy, in the case of *S. pombe* immediately followed by meiosis and sporulation, which for space issues we will not cover in this review.

### 4.1. Cell–cell fusion in *Saccharomyces cerevisiae*

Compared with the signalling and polarization mechanisms described above, the process of cell fusion is much less understood. This may be due to two main reasons: first, cell fusion can be studied only in mating pairs, and thus all the processes described above must occur normally to eventually reach this stage. This may preclude the identification of factors that function at several steps of the mating process, because their disruption would block the cell at an early stage. Second, almost all mutants identified to date exhibit only partial defects in cell fusion, typically blocking fusion in only 20–70% of all mating pairs, and this only if both mating partners are mutant, with a few exceptions. This suggests several pathways may redundantly mediate cell fusion, or the key components have not yet been identified. Nevertheless, genetic analysis has, over the years, identified a significant number of important players at both steps—cell wall digestion and plasma membrane fusion—of the fusion process.

Having come into contact by extending a projection towards each other, the two partner cells must engage in the fusion process. The timing of this engagement is probably critical and must be regulated: too early, the two cells would suffer from osmotic shock and lyse. How timing is sensed is unknown, but it has been proposed that cell fusion requires particularly high local levels of pheromone [100]. A role for pheromone signalling can also be inferred from the observation that the MAPK Fus3 is required for cell fusion [101]. In addition, it has been suggested that cells activate a protective pathway involving Pkc1 at early stages of mating prior to cell–cell contact, to antagonize cell wall reorganization until a mechanical signal owing to turgor pressure inactivates it to promote fusion [102].

A localized fusion machinery is essential for successful fusion, and so a large part of the polarization machinery is re-used for fusion. For instance, specific alleles of Cdc42 and its GEF Cdc24 have been identified that block cell fusion, but not earlier events [103–105], indicating the main polarization machinery also controls cell fusion. The actin cytoskeleton, which is essential for polarized growth and vesicle transport, probably also plays a specific role in cell fusion, although a direct role has not been demonstrated. Actin structures are reorganized during the mating process with the presence of actin dots at shmoo tips [106]. However, endocytosis, which in vegetative cells occurs in actin patches, does not appear essential for the mating process [107]. By contrast, actin cables and vesicle transport are required for fusion: tropomyosin *tpm1* mutants, in which actin cables are destabilized, increase the number of secretory vesicles at the shmoo site with apparent defects in cell shmooing and fusion [108]; similarly, deletion of the formin Bni1, which assembles actin cables, or of members of the polarisome that regulate its localization and/or activity, such as Spa2, led to fusion defects [109,110]; finally, the type V myosin Myo2 transports cell wall remodelling enzymes such as chitin synthase 3 as well as the MAPK scaffold Ste5 to the shmoo tips [60,111–113]. Mutants in the secretory pathway also strongly block cell fusion, even when inactivated in pre-zygotes and mated with wild-type partners [114], suggesting exocytosis is critical for cell fusion.

One important function of the polarization and actin apparatus is to promote the localization of fusion-specific factors critical for cell wall remodelling (figure 3*a*). In particular, Fus1, an O-glycosylated 1-pass plasma membrane protein [115–117], depends on Cdc42, its GEF Cdc24, and a late Golgi trafficking protein, Chs5, for localization at the shmoo tip and fusion site [103,104,111]. Fus1 specifically affects the fusion, as upon *fus1* deletion both partners are still able to sense, attract and grow mating projections towards each other, and is required for vesicle positioning and clustering at the fusion site [110]. In turn, Fus1 promotes the anchoring of a second fusion-specific factor, Fus2 [118]. However, Fus2 and Fus1 play additive functions, as complete fusion block is only achieved in double mutants, which arrest at a pre-zygote stage with cell wall material separating the two partner cells [110,116]. Fus2 is probably transported to the fusion site along actin cables, as its localization depends on the polarisome and the type-V myosin Myo2 [112,118]. It functions late during fusion, blocking pairs with vesicles tightly clustered at the zone of fusion [110]. Similar phenotype is observed for a specific *cdc42* allele, which displays defects only in cell fusion [105]. Fus2, which contains a putative Rho-GEF domain, in fact binds GTP-Cdc42 directly, suggesting it acts as a Cdc42 effector for fusion [105]. Fus2 also requires Rvs161, a BAR-domain protein best characterized for its function in endocytosis, but which functions here in an endocytosis-independent manner [107,118]. Remarkably, at the fusion site, Fus2 localizes as an expanding ring, and is proposed to remove cell wall remnants as fusion proceeds [118] (figure 3*a*).

Cell wall remodelling allows plasma membrane contact for fusion. Plasma membrane composition and dedicated transmembrane proteins are both critical for this latter process. Bioinformatic screens for transmembrane proteins, whose expression is induced by pheromone, revealed Prm1, which localizes at the fusion site [119,120] (figure 3*a*). Prm1 mutants degrade the cell wall between both partners as observed by electron microscopy, but cannot efficiently fuse their plasma membranes. Prm1 is a 4-pass plasma membrane protein, with two extracellular loops that, in the endoplasmic reticulum, assemble dimers stabilized by disulfide bonds [121,122]. Three observations suggest Prm1 is not the elusive cell–cell fusogen, but regulates the fusion process through distinct mechanisms: first, Prm1 conformation is distinct from known 1-pass transmembrane fusogens, such as SNARE proteins or viral fusogens: second, defective plasma membrane fusion in *prm1Δ* cells can cause cell lysis, which cannot be prevented by osmotic stabilization [123]; third, only 60 per cent of *prm1Δ* mating pairs are unable to fuse [119]. It has been proposed that Prm1 may promote the formation of a fusion pore through the insertion into the plasma membrane of the partner cell of a hydrophobic region present in its first extracellular loop, upon disulfide bond reduction [122]. Alternatively, Prm1 may form a molecular fence around the fusion pore to protect from membrane damage [124], a function that can in part be compensated by addition of Ca^2+^ in the medium, to promote repair mechanisms. Three other proteins are proposed to act for plasma membrane fusion: Fig1, a 4-pass transmembrane Ca^2+^ influx regulator, and Kex2, a Golgi-resident protease necessary for the proteolytic processing of alpha-factor, both act additively to Prm1 for membrane fusion [124,125] (figure 3*a*). Fus1, described above for its role in cell wall remodelling, has been implicated in membrane pore opening and expansion during cell fusion, although its specific function is unknown [126]. Finally, the pheromone receptors probably contribute to the fusion process, as they form heterotypic interactions able to bring membranes in close juxtaposition. However, specific mutations preventing this interaction block cell fusion with cell wall remaining at the cell–cell junction [127].

Plasma membrane fusion also depends on its composition. In pheromone-treated cells, the lipid bilayer at the shmoo tip is more condensed than the rest of the plasma membrane, an organization that depends on sphingolipids [128]. In particular, phosphatidylinositol 4,5-bisphosphate (PIP_2_) and ergosterols are enriched at the shmoo tip, and are required for Ste5 scaffold recruitment and MAPK activation [128,129]. Ergosterols are further enriched at the fusion site and deletion of enzymes involved in the late steps of ergosterol biosynthesis causes membrane fusion delays and defects [130,131], suggesting ergosterols may act as cofactors to concentrate some unknown component of the cell-fusion machinery. In summary, the process of cell–cell fusion depends on many protein and lipid factors acting at distinct steps, but the critical fusogen mediating plasma membrane fusion remains undiscovered.

### 4.2. Cell–cell fusion in *Schizosaccharomyces pombe*

The process of cell fusion has not received much attention in fission yeast. However, as for mating signalling and polarization, several observations suggest that considerable knowledge would be gained from studying cell fusion in this organism. In particular, the role of the actin cytoskeleton in cell fusion is more evident in *S. pombe*, as these cells express a specific pheromone-dependent actin nucleator, the formin Fus1 (entirely distinct from its *S. cerevisiae* homonym Fus1), essential for cell fusion. In addition, *fus1Δ* cells are fully fusion-deficient, suggesting the fusion machinery may be less redundant in *S. pombe* than *S. cerevisiae* [132].

Fus1 is targeted to the shmoo tip by its N-terminus and requires its actin nucleation activity to promote cell fusion [133,134]. Deletion of *fus1* disrupts actin localization at the shmoo tip and blocks mating pairs at the pre-zygote stage with an intact cell wall [133]. Tropomyosin and type-V myosin are also important for fusion: tropomyosin (Cdc8) localizes as a small dot at the fusion site, and has been suggested to organize a small F-actin organelle at the cell contact site [135]. Myo51, one of the two type-V myosins of fission yeast, also localizes in a dot-like structure at the fusion site [136] (figure 3*b*). As these are also involved in cell fusion in *S. cerevisiae*, it suggests the actin cytoskeleton is used similarly by both organisms, but in absence of a dedicated formin in *S. cerevisiae*.

*Schizosaccharomyces pombe* does not encode orthologues of either *S. cerevisiae* Fus1 or Fus2. There is also very little known on plasma membrane fusion: a single study described a role for Dni1, a close relative of Fig1, whose localization to the shmoo tip depends on formin Fus1 and lipid domains, to be implicated in a Ca^2+^-independent manner in plasma membrane and cell wall remodelling during fusion [137]. Prm1 is highly conserved in *S. pombe*, and appears essential for cell–cell fusion (figure 3*b*; O. Dudin & S. G. Martin 2012, unpublished data). The question of cell–cell fusion would merit more attention in fission yeast.

## 5. Beyond yeast

The main proteins involved in the mating pathways of these two simple yeast models are conserved and participate in important processes in response to external signal in other organisms. In higher eukaryotes, for instance, Cdc42 is involved both in axon specification and in dendrite development in response to growth factors in neurons [138], and promotes chemokine-induced T-cell polarity to allow migration of T cells [139]. The mechanisms controlling Ras activation have also been conserved during evolution. Indeed, in most eukaryotic cells, Ras proteins participate in signal transduction pathways that modulate gene expression in response to external signals and are mediated by the activation of MAPK cascades. In mammalian cells, Ras hyper-activation is often associated with tumour development, although oncogenic mechanisms are only partially understood. However, similarly to *pombe* Ras1, human Ras activates a conserved Raf–MAPK cascade to promote gene expression and induces cytoskeleton reorganization, which requires Rho family GTPases Cdc42 and Rac [140]. Finally, G-protein-coupled receptors regulate diverse biological processes in all eukaryotes and are the most targeted proteins in pharmacological design [141]. Thus, a deeper analysis of the downstream effectors of these transmembrane proteins in simple organisms can be helpful to understand more complex pathways in higher eukaryotes and to discover new therapeutic drugs.

The process of cell fusion also underlies several important developmental events, including fertilization, muscle fibre formation, placenta development and osteoclast formation. In very few cases have the bona fide fusogens been identified [142]. It is thus currently unclear whether mechanisms of cell–cell fusion will rely on conserved molecular machineries, similar to those underlying vesicle fusion. Nevertheless, yeast cell fusion bears similarities for instance to myoblast fusion, best studied in *Drosophila*. Here, a fusion-competent myoblast (FCM) migrates towards a founder cell. Upon contact and adhesion, a prominent actin structure, in this case dependent on Arp2/3 nucleation, forms in the FCM and recruits other factors for cell fusion [143]. The presence of a dedicated actin structure, one of the most conserved features of myoblast fusion, suggests a parallel with yeast. In addition, Cdc42 and its orthologue Rac1 have been implicated in cell fusion not only in yeast, but also in mouse and *Drosophila* myoblasts [144,145]. However, in contrast to yeast, the system is inherently asymmetric, with the actin structure forming only in the FCM and cell–cell interaction relying on heterotypic interactions. Except for the heterotypic interaction of the pheromone receptors reported in *S. cerevisiae* [127], yeast cell mating appears largely symmetric [146], with both mating types assembling a fusion machinery, although it has been suggested that *S. pombe* M cells ‘take the initiative’ for mating [147]. Future work may reveal the extent of the analogies between diverse types of cell fusions.

As we hope will be clear from this review, we now understand in great detail some of the molecular connections underlying the response to pheromones. However, many molecular questions remain wide open: what are the molecular connections between pheromone sensing and signalling in fission yeast? How did such divergent connections evolve in the ascomycete lineage and beyond? What are the initial steps that allow the orientation of the polarization apparatus in response to pheromones? What is the molecular nature of the apparatus mediating cell–cell fusion? Beyond the single cell response, how groups of cells interact at a system level also raises many questions: how is a pheromone landscape shaped in a cell population? How do cells make a ‘choice’ for one partner when presented with many options? How is this choice sustained during polarized growth? How are other potential partners for a mating pair ‘discouraged’? Continued investigation using these two highly divergent yeast species will undoubtedly reveal novel insights into these and other fascinating questions.

## 6. Acknowledgements

We are grateful to Felipe Bendezú and Serge Pelet for their suggestions. Research in S.G.M.'s laboratory is supported by a Swiss National Science Foundation Research grant (no. 31003A_138177) and a European Research Council Starting grant (no. 260493).
